# Systematic analysis of the kalimantacin assembly line NRPS module using an adapted targeted mutagenesis approach

**DOI:** 10.1002/mbo3.326

**Published:** 2015-12-15

**Authors:** Birgit Uytterhoeven, Kenny Appermans, Lijiang Song, Joleen Masschelein, Thomas Lathouwers, Chris W. Michiels, Rob Lavigne

**Affiliations:** ^1^ Laboratory of Gene TechnologyKU LeuvenKasteelpark Arenberg 21 box 2462HeverleeB‐3001Belgium; ^2^ Department of ChemistryUniversity of WarwickGibbet HillCoventryCV4 7ALUnited Kingdom; ^3^ Centre for Food and Microbial TechnologyKU LeuvenKasteelpark Arenberg 23 box 2457HeverleeB‐3001Belgium; ^4^ Present address: Kenny AppermansCentre for Microbial and Plant geneticsKasteelpark Arenberg 20, box 2460HeverleeB‐3001Belgium

**Keywords:** Adenylation domain, kalimantacin, ligation independent cloning, specificity‐conferring code

## Abstract

Kalimantacin is an antimicrobial compound with strong antistaphylococcal activity that is produced by a hybrid *trans*‐acyltransferase polyketide synthase/nonribosomal peptide synthetase system in *Pseudomonas fluorescens* BCCM_ID9359. We here present a systematic analysis of the substrate specificity of the glycine‐incorporating adenylation domain from the kalimantacin biosynthetic assembly line by a targeted mutagenesis approach. The specificity‐conferring code was adapted for use in *Pseudomonas* and mutated adenylation domain active site sequences were introduced in the kalimantacin gene cluster, using a newly adapted ligation independent cloning method. Antimicrobial activity screens and LC‐MS analyses revealed that the production of the kalimantacin analogues in the mutated strains was abolished. These results support the idea that further insight in the specificity of downstream domains in nonribosomal peptide synthetases and polyketide synthases is required to efficiently engineer these strains *in vivo*.

## Introduction

**Figure 1:**
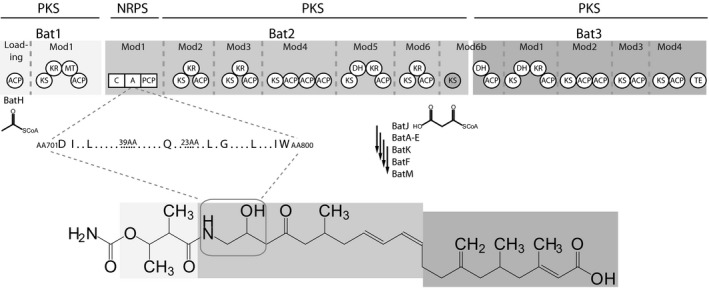
Biosynthetic pathway and structure of kalimantacin. Biosynthesis of kalimantacin is initiated in the loading module by the *trans*‐acting acyltransferase BatH, which loads an acetyl‐CoA unit onto the first ACP. A second *trans*‐AT, BatJ, selectively delivers malonyl‐CoA to the 11 remaining PKS‐modules in the biosynthetic pathway. The NRPS‐module in Bat2 incorporates a glycine residue into the growing intermediate. BatA‐E, BatK, BatF, and BatM perform several on‐ and post‐PKS tailoring reactions to produce the final bioactive compound. KS, ketosynthase; KR, ketoreductase; DH, dehydratase; ACP, acyl carrier protein; MT, methyltransferase; C, condensation domain; A, adenylation domain; PCP, peptidyl carrier protein; TE, thioesterase; AA, amino acid.

Nonribosomal peptides (NRPs) and polyketides (PKs) are two major classes of secondary metabolites with important therapeutic properties, such as antibiotic, antifungal, immunosuppressive, or antitumor activities. They are produced in bacteria and fungi by multimodular enzymatic assembly lines, known as nonribosomal peptide synthetases (NRPSs) and polyketide synthases (PKSs), respectively (Finking and Marahiel 2004; Smith and Tsai 2007). Due to similarities in their modular organization and biosynthetic strategies, PKSs and NRPSs are able to assemble into hybrid multienzyme complexes (Du et al. 2001). Kalimantacin, an antibiotic produced by *Pseudomonas fluorescens* BCCM_ID9359, is produced by such a hybrid NRPS‐PKS system and its biosynthetic pathway has been fully elucidated (Fig. 1) (Mattheus et al. 2010a). The kalimantacin enzymatic assembly line is composed of three polypeptides (Bat1, Bat2, and Bat3), harboring 11 *trans*‐acyltransferase (AT) PKS modules and one NRPS module. The latter incorporates glycine into the PK backbone and is situated in the first module of Bat2 (Bat2mod1). During and after assembly line biosynthesis, kalimantacin is modified by several *trans*‐acting tailoring enzymes (Fig. 1) (Mattheus et al. 2010a). Kalimantacin exerts strong antagonistic activity against staphylococci (MIC 0.05 *μ*g/mL) and has moderate activity against enterobacteria (MIC 1–10 *μ*g/mL). It acts by inhibiting the trans‐2‐enoyl‐ acyl carrier protein (ACP)‐reductase (FabI), an enzyme responsible for the final step in bacterial fatty acid biosynthesis (Mattheus et al. 2010b).

Nonribosomal peptide synthetases (NRPS) modules typically consist of a condensation (C) domain for peptide bond formation, an adenylation (A) domain for selection and activation of the cognate amino acid (AA) building block and a thiolation (T) domain or peptidyl carrier protein (PCP) that covalently tethers the growing intermediate to the enzyme complex (Finking and Marahiel 2004). NRPs exhibit a high structural diversity, which is partly determined by the substrate specificity of the A domains (Lautru and Challis 2004). Aside from the 22 proteinogenic AA, A domains can additionally introduce nonproteinogenic AAs and *α*‐hydroxy acids as well as nonsubstituted carboxylic acids. The substrate‐binding pocket of A domains is lined by 10 AA residues that determine the nature of the AA that is incorporated into the NRP backbone. This specificity‐conferring code can be bioinformatically extracted and enables the structure prediction of new NRPs based on the genomic arrangement of the corresponding biosynthetic genes (Stachelhaus et al. 1999; Challis et al. 2000; Röttig et al. 2011). This has already been exploited to rationally alter the specificity of A domains by targeted mutagenesis. So far, only relatively small changes in A domain specificity, for example, Asp to Asn, Glu to Gln, Phe to Tyr, have been achieved in *in vitro* experiments with purified mutant A domains (Stachelhaus et al. 1999; Eppelmann et al. 2002; Stevens et al. 2006). Recently, the Phe‐incorporating A domain of gramicidin synthetase (GrsA) has been successfully re‐engineered to introduce azide‐ and alkyne‐substituted AA (Kries et al. 2014). Attempts to drastically change the substrate specificity have resulted in a decrease in the A domain activity (Chen et al. 2009). *In vivo* efforts to create NRP analogues with variations in AA composition by targeted mutagenesis of A domains appear to be even more challenging (Eppelmann et al. 2002; Uguru et al. 2004; Han et al. 2012; Thirlway et al. 2012). In this study, we attempted to rationally and systematically change the specificity of the glycine‐incorporating A domain in the NRPS module of the kalimantacin biosynthetic assembly line by using a new targeted mutagenesis approach and a specificity‐conferring code designed for use in *Pseudomonas*. The functionality and specificity of the mutated A domains were subsequently analyzed by ultrahigh‐resolution LC‐MS analysis of culture supernatants.

## Experimental Procedures

### Bacterial strains and culture conditions

*Pseudomonas fluorescens* BCCM_ID9359 was used for wild‐type kalimantacin production and was grown in tryptose broth (Merck) or tryptose agar (tryptose broth supplemented with 1.5% w/v agar) at 30°C (Mattheus et al. 2010a). *Escherichia coli* Top10 (Invitrogen^™^, Carlsbad, CA) was used for amplification of plasmids and cloning purposes, whereas *E. coli* S17‐1 (Simon et al. 1983) was used for conjugational transfer to *P. fluorescens* BCCM_ID9359. All *E. coli* strains were grown in lysogeny broth (LB) or LB agar (LB broth with 1.5% w/v agar) at 37°C. Strains used in this study are listed in Table S2.

### Construction of adenylation domain mutants

Plasmids and primers used in this study are listed in Table S2. First 5′ and 3′ flanking regions of the adenylation domain active site sequence were amplified with tailed primers introducing restriction sites and these fragments were restricted and ligated into pUC18 using T4 DNA ligase (Thermo scientific) following the manufacturer's protocols. The sequence of this construct was verified as described below. The mutated active site fragments were chemically synthesized at Integrated DNA Technologies (Haasrode, Belgium) and amplified for ligation independent cloning by *pfu* DNA polymerase (Thermo scientific, Waltham, MA).

The ligation independent cloning method was adapted from Thieme et al. (2011). First, the pUC18 plasmid with 5′ and 3′ fragment was treated with PstI. A mixture of this linearized plasmid, the amplified synthetic DNA fragment, and T4 DNA polymerase was incubated for 5 min at 25°C, immediately followed by transformation of *E. coli* Top10. The sequence of the resulting plasmids was verified by sequence analysis. The complete 5′–3′ construct with adapted adenylation domain sequence was then transferred from pUC18 to suicide plasmid pAKE604 (El‐Sayed et al. 2003) by regular restriction and ligation and again verified by sequence analysis.

To easily detect mutated active site introduction, a plasmid containing the 5′ and 3′ flanking sequences was constructed, flanking the gentamicin resistance gene. All fragments were amplified by tailed primers introducing restriction sites (as listed in Table S2). After restriction, they were ligated using T4 DNA ligase. The construct was verified by sequence analysis. This construct was introduced in the genome of *P. fluorescens* BCCM_ID9359 by biparental conjugation with *E. coli* S17‐1. Both strains were grown to late exponential phase and mixed on a sterile 0.45 *μ*m Millipore filter on tryptose agar. After overnight incubation, the filter was washed with 1 mL 0.9% sodium chloride and plated on tryptose agar supplemented with kanamycin and triclosan to select for vector integration by a first crossover event. Next, colonies were incubated overnight in tryptose broth without any selection to enable vector excision and plated in serial dilutions on tryptose agar with 5% w/v sucrose. Colonies were then checked by replica plating on tryptose agar with gentamicin and tryptose agar with kanamycin. Gentamicin‐resistant, but kanamycin‐sensitive clones were verified by PCR with primers external of the 5′ and 3′ flanking region and the amplified region was then verified by sequence analysis.

Finally, to introduce the mutated active site sequence, the gentamicin‐resistant strain was used for another round of biparental conjugation with the mutated active site constructs in pAKE604 in *E. coli* S17‐1. The same strategy was used as described above, selecting at the end for gentamicin‐sensitive clones. Again, the genome of the strain was verified by PCR with the external primers, followed by sequencing of this PCR product.

### Sequence analysis of plasmids and genomic regions

Plasmids or PCR fragments of genomic regions were used in a Sanger sequencing reaction with Big Dye terminator mix (Applied biosystems, Waltham, MA) and the resulting mixture was purified by ethanol precipitation. Samples were run on an ABI 3130 genetic analyzer (Applied biosystems) and analyzed using Sequencer 4.8 software (GeneCodes Inc, Ann Arbor, MI).

### Bioassay of strains and extracts

After large‐scale fermentation in 500 mL cultures, kalimantacin and analogues were extracted with chloroform, as described by Mattheus et al. (2010a). The activity of the extracts was tested by spotting 2 *μ*L of the extract on a lawn of *Staphylococcus aureus* ATCC27661 in stationary phase, diluted to an optical density at 600 nm of 0.03. Results were recorded after 12 h of incubation at 37°C. The activity of the mutant strains was evaluated by spotting 2 *μ*L of an overnight culture on a similar lawn of *S. aureus* ATCC27661. Plates were incubated for 48 h at 16°C, followed by 12 h at 37°C.

### LC/MS analysis of kalimantacin production

After 24 h at 16°C, followed by 12 h at 4°C, the pH of 50 mL fermentation cultures was adjusted to pH 10 with formic acid. After centrifugation, 2 *μ*L of the supernatant was analyzed directly with LC‐MS (column: Agilent Zorbax Eclipse RP‐C18, 100 × 2.1 mm, 1.8 *μ*m). A Bruker MaXis Impact Q‐TOF mass spectrometer coupled with a Dionex 3000RS UHPLC was used. Mobile phases consist of A, water with 0.1% formic acid, and B, acetonitrile with 0.1% formic acid. After the initial 5 min of isocratic run at 5% B, a gradient of 5B–100% B in 18 min, then isocratic at 100% B for 5 min was employed with flow rate at 0.2 mL/min; UV was set at 210 nm. The mass spectrometer was operated in electrospray positive mode with a scan range of 50–3000 *m/z*. Source conditions are: end plate offset at −500 V; capillary at −4500 V; nebulizer gas (N2) at 1.4 bar; dry gas (N_2_) at 8 L/min; dry temperature at 180°C. Ion transfer conditions as, ion funnel 1 RF at 200 Vpp; ion funnel 2 RF at 200 Vpp, hexapole RF at 200 Vpp; quadruple ion energy at 5 ev, quadrupole low mass set at 55 *m/z*; collision energy at 5.0 ev; collision RF ramping from 800 to 1500 Vpp; transfer time set from 100 to 155 *μ*sec; pre‐Pulse storage time set at 5 *μ*sec. Calibration was done with sodium formate (10 m mol/L) through a loop injection of 20 *μ*L of standard solution at the beginning of each run.

## Results

### Generation of a *Pseudomonas*‐specific specificity‐conferring code

Research into the specificity‐conferring code of A domains has revealed that genus‐specific variations exist at specific positions in the code (Challis et al. 2000), which hint at “wobble‐like” positions (Stachelhaus et al. 1999). Therefore, we hypothesized that the specificity of mutated A domains can be improved by the use of a genus‐specific code. A literature wide study was performed, searching for NRPSs with characterized products in *Pseudomonas* spp. and the different specificity‐conferring codes of the A domains were extracted using NRPSpredictor2 (Table S1) (Röttig et al. 2011). Since all of these codes were derived from *Pseudomonas* sequences, they were considered *Pseudomonas* specific, or at least compatible. For each position in the specificity‐conferring code, the most commonly encountered AA was selected and preference was given to AAs present in A domains from NRPSs in *P. fluorescens* spp. As such, 10 specificity‐conferring codes were designed with the aim of replacing Gly in the final structure of kalimantacin with 10 alternative AAs, namely Ala, Leu, Val, Ser, Cys, Arg, Asp, Gln, Phe, and Pro, representing different natural AA classes (Table 1).

**Table 1:** Overview of adapted specificity‐conferring codes for use in *Pseudomonas* **.** AA positions and the corresponding specificity‐conferring codes of the Phe‐incorporating A domain in GrsA and the Gly‐incorporating A domain in Bat2mod1 are illustrated. Codes were predicted by comparative analysis of A domain from different *Pseudomonas* NRPSs

		AA1	AA2	AA3	AA4	AA5	AA6	AA7	AA8	AA9	AA10
		235	236	239	278	299	301	322	330	331	517
Phe	grsA	D	A	W	T	I	A	A	I	C	K
Gly	bat2	D	I	L	Q	L	G	L	I	W	K
		701	702	705	744	767	769	791	799	800	–
Ala		D	L	Y	N	N	A	L	T	Y	K
Leu		D	A	W	F	L	G	N	V	V	K
Val		D	A	L	W	I	G	G	T	F	K
Ser		D	V	W	H	M	S	L	V	D	K
Gln		D	A	W	Q	V	G	V	V	D	K
Cys		D	L	Y	N	L	S	P	I	W	K
Arg		D	V	A	D	V	G	A	I	D	K
Phe		D	A	P	I	M	G	G	T	C	K
Asp		D	S	W	K	L	G	V	V	D	K
Pro		D	V	Q	Y	I	A	H	V	V	K

### Ligation independent cloning enables efficient introduction of mutated active site fragments

**Figure 2:**
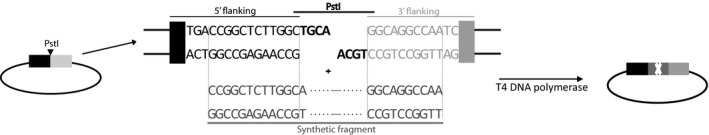
Ligation independent cloning strategy. Flanking regions (350 bp and 500 bp) of the 10 AA containing active site were amplified from genomic DNA with tailed primers, introducing restriction sites that enabled restriction and ligation into the pUC18. After restriction with PstI, a mixture of linear plasmid DNA, amplified synthetic DNA fragment and T4 DNA polymerase was prepared, as proposed by Thieme *et al*. (2011). The mixture was incubated at 25°C for 5 min, and subsequently used for transformation of *E. coli* Top10 cells (Invitrogen^™^). Correct constructs were obtained with very high efficiencies (80–95%).

To introduce these adapted specificity‐conferring codes in the A domain of Bat2mod1, the positions of the 10 AAs in Bat2mod1 that comprise the original code were identified by sequence alignments with the archetypic GrsA (Stachelhaus et al. 1999; Challis et al. 2000). Taking into account the codon usage of *Pseudomonas protegens* Pf5, DNA fragments encoding the altered specificity‐conferring codes were designed. The specificity of these A domains was verified with NRPSpredictor2, using the nearest neighbor method (Röttig et al. 2011). All codes were predicted to introduce the intended AA with a score of 90%, except for Gln, which would be introduced with a predicted score of 60%. Chemically synthesized gene fragments containing the correct mutations were cloned into a pUC18 (Invitrogen^™^) plasmid, containing the two flanking regions (±350 base pairs (bp) and 500 bp) of the mutated active site, using an adapted ligation independent cloning approach. An overlap of 12 bp between the flanking fragments and the synthetic DNA fragment allowed efficient ligation independent cloning, as illustrated in Figure 2 (Thieme et al. 2011). Following sequence analysis, the complete fragment, comprising the 5′ and 3′ flanking regions and the mutated active site sequence, was transferred to suicide vector pAKE604 (El‐Sayed et al. 2003) via regular restriction and ligation.

To facilitate the introduction of the mutated active site sequences in *bat2* in *P. fluorescens* BCCM_ID9359, the 400 bp genomic fragment, targeted for mutation, was first replaced by a gentamicin resistance cassette via double homologous recombination. Next, the vector carrying the mutated active site and flanking regions was introduced into this gentamicin‐resistant *P. fluorescens* mutant via conjugation and a first crossover event, enabling exchange of the resistance cassette with the active site via a second crossover event.

PCR and sequence analysis of the constructed plasmids revealed a very high efficiency for the ligation‐independent cloning method (80–95%). Recombination of these plasmids and the gentamicin‐resistant strains, using 5′ 350 bp and 3′ 500 bp homologous regions, resulted in one gentamicin‐sensitive clone out of five tested clones. Sequence analysis of these strains indicated that all gentamicin‐sensitive strains had incorporated the mutated active site sequence without errors. As a positive control, the WT active site sequence was also reintroduced in the gentamicin‐resistant strain.

### Bioassay and LC‐MS analyses show the absence of production of kalimantacin analogues

**Figure 3:**
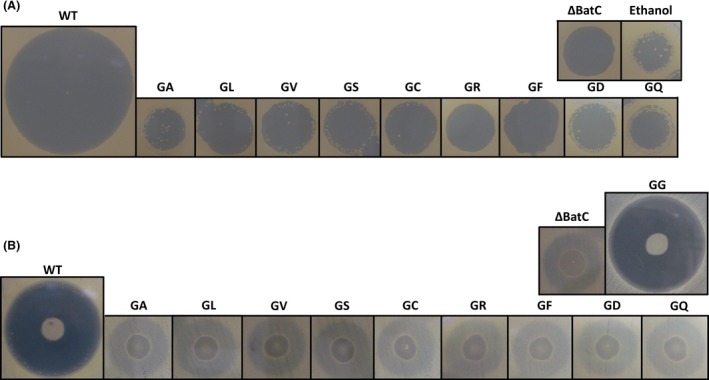
Bioassay with kalimantacin and analogues. (A). Halo formation by extracts of *Pseudomonas fluorescens* BCCM_ID9359 wild‐type and mutant strains. 2 μL of crude extract was spotted on a lawn of *S. aureus* ATCC27661 (OD_600_ = 0.03) and incubated overnight at 37°C. (B). Halo formation by the wild‐type and engineered strains. 2 μL of an overnight culture was spotted on a lawn of *S. aureus* ATCC27661 (OD_600_ = 0.03). Incubation at 16°C for 48 h allowed for growth of *P. fluorescens* BCCM_ID9359 (and mutant strains) and production of kalimantacin or analogues, followed by incubation at 37°C for 8 h for the growth of the lawn of *S. aureus*. WT, wild‐type strain/extract; GA, kalimantacin with alanine instead of glycine; GL, kalimantacin with leucine instead of glycine; GV, kalimantacin with valine instead of glycine; GS, kalimantacin with serine instead of glycine; GC, kalimantacin with cysteine instead of glycine; GR, kalimantacin with arginine instead of glycine; GF, kalimantacin with phenylalanine instead of glycine; GD, kalimantacin with aspartic acid instead of glycine; GQ, kalimantacin with glutamine instead of glycine; GG, kalimantacin with glycine after reintroduction of the wild‐type sequence in the gentamicin‐resistant strain.

Bioassays with the mutant strains and the crude extracts of large‐scale fermentations on a lawn of *S. aureus* ATCC27661 cells revealed a clear decrease in activity for all of the mutants, comparable to the activity of the *batC* deletion mutant, which no longer produces kalimantacin, and its extract (Mattheus et al. 2010a). The positive control showed WT activity again (Fig. 3). To determine if the decrease in activity is due to low or abolished antibiotic production or to production of an inactive kalimantacin analogue, culture supernatants were analyzed by ultrahigh‐resolution LC‐MS. In the supernatant of the WT sample, the *m/z* value corresponding to the WT mass could be easily detected. The noise level was in the 100–200 counts range. However, in the supernatant of the mutated strains, no metabolites with *m/z* values corresponding to kalimantacin or kalimantacin analogues with the expected AA substitutions could be detected (Fig. S1, S2).

## Discussion and Conclusion

As a proof of concept, a *Pseudomonas*‐specific code was created in this article for A domains. Since, as far as we know, no specificity‐conferring codes for other genera have been published, it is difficult to compare this code with codes in other genera. However, when comparing the specificity‐conferring code of the archetypic Phe‐incorporating module of GrsA (DAWTIAAICK) with the presented code for use in *Pseudomonas* (DAPIMGGTCK), the difference between both codes is remarkable, with six out of 10 AA differing. Two of these six AA, at GrsA positions 301 and 322, are rather small changes, going from two times Ala to two times Gly, thus removing two small neutral groups out of the binding pocket. The other changes seem more drastic, as polar groups change into neutral groups or vice versa. It should be noted that changes do not necessarily imply drastic differences between codes in different genera. If the Pro‐incorporating sequence is compared to the consensus sequence proposed by Stachelhaus and colleagues, only one of all AA is changed (from Tyr to Leu). As it goes with codon optimization, it is possible that optimizing the specificity‐conferring code, as is done here for *Pseudomonas,* improves the yield, but does not necessarily mean that a code from another organism does not give any result at all.

The bioassay of the positive control, showing wild‐type activity again, proves the robustness of the method presented. Overall, this method is very efficient for the *in gene* replacement of active site sequences. Furthermore, chemical synthesis of the mutated DNA fragments circumvents an elaborate targeted point mutation approach. Therefore, this method is particularly suited for systematic *in vivo* analyses of mutated A domains.

All bioassays show a residual activity of both mutant strains and extracts, comparable to the *bat*C deletion mutant. Since LC‐MS analyses show no detectable amounts of kalimantacin and it seems unlikely that all mutants produce the same kalimantacin intermediate as the *bat*C knockout strain, this activity can only be explained by another, unrelated secondary metabolite produced by the *P. fluorescens* strain, with low but distinguishable activity against *S. aureus* ATCC27661. Additional bioassays against a collection of gram‐positive and gram‐negative strains showed that all mutant strains and the wild‐type strain have a similar activity against different *Pseudomonas* strains (data not shown), indicating that other antibacterial secondary metabolites are indeed produced by *P. fluorescens* BCCM_ID9359.

There are a number of possible reasons why the kalimantacin biosynthetic assembly lines harboring the mutated A domains are no longer functional. First, the *Pseudomonas*‐specific code might not be sufficiently adapted. However, all the critical conserved AA residues as proposed by Challis and colleagues (Challis et al. 2000) are present in each of the designed specificity‐conferring codes. *In vitro* analysis of the specificity of the engineered A domains could shed more light on this. Furthermore, working in an *in vivo* system, downstream domains might not accept the alternative biosynthetic intermediates. Indeed, it has recently been shown that ketosynthase (KS) domains in *trans*‐AT PKSs show specificity to the growing intermediate as well (Jenner et al. 2013). In addition, a recent paper by Kohlhaas and coworkers on the specificity in hybrid *trans*‐AT PKS/NRPS clusters confirms that the AA‐accepting KS domains possess a high specificity for NRPS‐derived intermediates (Kohlhaas et al. 2013). In the kalimantacin cluster, this is expected to be the case for the KS domain of module 2 in Bat2. Engineering the substrate selectivity of this KS domain or replacing it with a KS domain that has an increased substrate tolerance could potentially resolve this issue. Not only the downstream domains could block synthesis, different studies have shown that the acceptor site of the C domain could also possess substrate selectivity (Belshaw et al. 1999; Ehmann et al. 2000). As such, it is possible that the A domain is active, while the C domain is not accepting the alternative building block. This selectivity of the C domain was recently demonstrated by Calcott et al. (2014) in the pyoverdine synthetase PvdD in *P. aeruginosa*, and could also explain why the *in vivo* system in the *P. fluorescens* strain in this study is blocked.

In this article, we present an efficient method to introduce mutated active site sequences in the biosynthetic gene clusters. However, more insight is needed in the specificity of upstream and downstream domains to rationally engineer A domains in hybrid *trans*‐AT PKS/NRPS clusters.

## Conflict of Interest

None declared.

## Supporting information


**Figure S1.** Extracted ion chromatograms of culture supernatants from wild‐type *P. fluorescens* BCCM_id9359 and mutants at expected *m/z* values
**Figure S2**. Extracted ion chromatograms at m/z value of WT of culture supernatants from wild‐type *P. fluorescens* BCCM_ID9359 and mutants.
**Table S1**. An overview of the specificity‐conferring codes of A domains from different *Pseudomonas* strains used to create a Pseudomonas‐specific code.
**Table S2.** Overview of the strains, plasmids and primers used in this study.
