# Supplementary material for: Systematic analysis of the kalimantacin assembly line NRPS module using an adapted targeted mutagenesis approach
Source: Microbiologyopen. 2015 Dec 15;5(2):279–86. doi: 10.1002/mbo3.326 (PMC4831472; doi:10.1002/mbo3.326)
Supplement: Supplementary file 1 — Figure S1. Extracted ion chromatograms of culture supernatants from wild‐type P. fluorescens BCCM_id9359 and mutants at expected m/z values Figure S2. Extracted ion chromatograms at m/z value of WT of culture supernatants from wild‐type P. fluorescens BCCM_ID9359 and mutants. Table S1. An overview of the specificity‐conferring codes of A domains from different Pseudomonas strains used to create a Pseudomonas‐specific code. Table S2. Overview of the strains, plasmids and primers used in this study. [file MBO3-5-279-s001.docx]

**Supplementary table 1. An overview of the specificity-conferring codes of A domains from different Pseudomonas strains used to create a Pseudomonas-specific code.** NRPSs with characterized products from Pseudomonas spp. were selected and the respective specificity-conferring codes of the A domains were extracted using NRPSpredictor2 (column 3). For every specificity-conferring code, the type of side chain (column 1), the AA specificity (column 2), the strain (column 4), the name of the compound (column 5), the module (column 6) and the Genbank accession number (column 7) are described.

| **Side chain** | **Incorporated AA** | **10 AA code** | **Strain** | **Compound** | **Module** | **GenBank number** |
| --- | --- | --- | --- | --- | --- | --- |
| **reference** | Phe | DAWTIAAICK | *Bacillus brevis* | gramicidin S | GrsA - PheA | CAA33603.1 |
| **neutral** | Gly | DILQLGLIWK | *P. fluorescens* BCCM_ID9359 | batumin | Bat2 mod2 | ADD82940.1 |
|  | Ala | DLYNNALTYK | *P. syringae* pv. *syringae* | syringopeptin | SypA mod5 | AAF99707.2 |
|  | Ala | DLYNNALTYK | *P. syringae* pv. *syringae* | syringopeptin | SypB mod6 | AAO72424.1 |
|  | Ala | DLYNNALTYK | *P. syringae* pv. *syringae* | syringopeptin | SypB mod10 | AAO72424.1 |
|  | Ala | DLYNNALTYK | *P. syringae* pv. *syringae* | syringopeptin | SypC mod13 | AAO72425.1 |
|  | Ala | DLYNNALTYK | *P. syringae* pv. *syringae* | syringopeptin | SypC mod17 | AAO72425.1 |
|  | Ala | DLYNNALTYK | *P. syringae* pv. *syringae* | syringopeptin | SypC mod12 | AAO72425.1 |
|  | Ala | DLYNNALTYK | *P. syringae* pv. *syringae* | syringopeptin | SypC mod19 | AAO72425.1 |
|  | Leu | DAWFLGNVVK | *P. fluorescens* SS101 | massetolide A | MassA mod1 | ABH06367.1 |
|  | Leu | DAWFLGNVVK | *P. fluorescens* SS101 | massetolide A | MassB mod3 | ABH06368.2 |
|  | leu | DAWFLGNVVK | *P. fluorescens* SS101 | massetolide A | MassC mod1 | ABH06369.2 |
|  | Leu | DAWFLGNVVK | *Pseudomonas* sp. DSS73 | amphisin | AmsY | CAC94829.1 |
|  | Leu | DAWFLGNVVK | *P. putida* PCL1445 | putisolvin | PsoA mod1 | ABW17375.1 |
|  | Leu | DAWFLGNVVK | *P. putida* PCL1445 | putisolvin | PsoB mod3 | ABW17376.1 |
|  | Leu | DAWFLGNVVK | *P. putida* PCL1445 | putisolvin | PsoC mod10 | ABW17377.1 |
|  | Leu | DAWFLGNVVK | *P. fluorescens* SBW25 | viscosin | ViscA mod1 | CAY50416.1 |
|  | Leu | DAWFLGNVVK | *P. fluorescens* SBW25 | viscosin | ViscB mod5 | CAY48788.1 |
|  | Leu | DAWFLGNVVK | *P. fluorescens* SBW25 | viscosin | ViscC mod7 | CAY48789.1 |
|  | Leu | DAWFLGNVVK | *P. syringae* pv. *tomato* str. DC3000 | syringafactin | SyfA mod1 | AAO56328.1 |
|  | Leu | DAWFLGNVVK | *P. syringae* pv. *tomato* str. DC3000 | syringafactin | SyfA mod2 | AAO56328.1 |
|  | Leu | DAWFLGNVVK | *P. syringae* pv. *tomato* str. DC3000 | syringafactin | SyfB mod4 | AAO56329 |
|  | Leu | DAWFLGNVVK | *P. syringae* pv. *tomato* str. DC3000 | syringafactin | SyfB mod7 | AAO56329 |
|  | Leu | DAWFLGNVVK | *P. syringae* pv. *tomato* str. DC3000 | syringafactin | SyfB mod8 | AAO56329 |
|  | Leu | DAWFLGNVVK | *P. protegens* Pf-5 | orfamide | OfaA mod1 | AAY91419.3 |
|  | Leu | DAWFLGNVVK | *P. protegens* Pf-5 | orfamide | OfaB mod5 | AAY91420.2 |
|  | Leu | DAWFLGNVVK | *P. protegens* Pf-5 | orfamide | OfaC mod7 | AAY91421.3 |
|  | Leu | DAWFLGNVVK | *P. protegens* Pf-5 | orfamide | OfaC mod8 | AAY91421.3 |
|  | Leu | DAWFLGNVVK | *Pseudomonas* sp. MIS38 | arthrofactin | ArfA mod1 | BAC67534.2 |
|  | Leu | DAWFLGNVVK | *Pseudomonas* sp. MIS38 | arthrofactin | ArfB mod4 | BAC67535.1 |
|  | Leu | DAWFLGNVVK | *Pseudomonas* sp. MIS38 | arthrofactin | ArfB mod5 | BAC67535.1 |
|  | Leu | DAWFLGNVVK | *Pseudomonas* sp. MIS38 | arthrofactin | ArfC mod7 | BAC67536.1 |
|  | Val/Leu, Ile | DALFIGGTFK | *P. putida* PCL1445 | putisolvin | PsoC mod11 | ABW17377.1 |
|  | Val | DALWIGGTFK | *P. syringae* pv. *syringae* | syringopeptin | SypA mod4 | AAF99707.2 |
|  | Val | DALWIGGTFK | *P. syringae* pv. *syringae* | syringopeptin | SypA mod3 | AAF99707.2 |
|  | Val | DALWIGGTFK | *P. syringae* pv. *syringae* | syringopeptin | SypB mod7 | AAO72424.1 |
|  | Val | DALWIGGTFK | *P. syringae* pv. *syringae* | syringopeptin | SypB mod8 | AAO72424.1 |
|  | Val | DALWIGGTFK | *P. syringae* pv. *syringae* | syringopeptin | SypC mod11 | AAO72425.1 |
|  | Val | DALWIGGTFK | *P. putida* PCL1445 | putisolvin | PsoB mod7 | ABW17376.1 |
|  | Val | DALWIGGTFK | *P. fluorescens* SBW25 | viscosin | ViscB mod4 | CAY48788.1 |
|  | Val | DALWMGGTFK | *P. entomophila* L48 | entolysin | EtlB mod4 | CAK15815.1 |
|  | Val | DALWMGGTFK | *P. entomophila* L48 | entolysin | EtlC mod11 | CAK15815.1 |
|  | Val | DALWMGGTFK | *P. entomophila* L48 | entolysin | EtlB mod7 | CAK15815.1 |
|  | Val | DALFLGGTFK | *P. fluorescens* Pf-5 | orfamide | OfaC mod10 | AAY91421.3 |
|  | Val | DAMFIGGTFK | *P. syringae* pv. *tomato* str. DC3000 | syringafactin | SyfB mod6 | AAO56329 |
|  | Val | DAFWLGCVFK | *P. syringae* pv *syringae* B301D-R | syringolin | SylC mod1 | CAD70194.1 |
| **sulfur** | Cys | DLYNLSPIWK | *P. aeruginosa* PAO1 | pyocheline | PchF | AAC83657.1 |
|  | Cys | DLFNLSLIWK | *P. aeruginosa* PAO1 | pyocheline | PchE | AAC83656.1 |
| **basic** | Arg | DVADVGAIDK | *P. syringae* pv. *syringae* | syringomycin | SyrE module 5 | AAC80285.1 |
| **aromatic** | Phe | DAPIMGGTCK | *P. syringae* pv. *syringae* | syringomycin | SyrE module 6 | AAC80285.1 |
| **acid** | Asp, Asn (Glu, Gln) | DMKDLGMVDK | *P. syringae* pv. *syringae* | syringomycin | SyrE module 8 | AAC80285.1 |
|  | Asp | DSWKLGVVDK | *Pseudomonas* sp. MIS38 | arthrofactin | ArfA mod2 | BAC67534.2 |
|  | Asp | DSWKLGVVDK | *Pseudomonas* sp. MIS38 | arthrofactin | ArfC mod11 | BAC67536.1 |
| **amino** | Gln | DAWQVGVVDK | *P. entomophila* L48 | entolysin | Pseen3045 mod3 | CAK15815.1 |
|  | Gln | DAWQVGVVDK | *P. entomophila* L48 | entolysin | Pseen3045 mod6 | CAK15815.1 |
|  | Gln | DAWQVGVVDK | *P. entomophila* L48 | entolysin | Pseen3045 mod9 | CAK15815.1 |
|  | Gln | DAWQVGVVDK | *P. syringae* pv. *tomato* str. DC3000 | syringafactin | SyfA mod3 | AAO56328.1 |
|  | Gln | DAWQVGVVDK | *P. putida* | putisolvin | PsoB mod5 | ABW17376.1 |
| **alcohol** | Ser | DLWHLSLIDK | *P. syringae pv. syringae* | syringomycin | SyrE module 1&2 | AAC80285.1 |
|  | Ser | DVWHMSLVDK | *P. fluorescens* SS101 | massetolide A | MassB mod4 | ABH06368.2 |
|  | Ser | DVWHMSLVDK | *P. fluorescens* SS102 | massetolide A | MassC mod2 | ABH06369.2 |
|  | Ser | DVWHMSLVDK | *P. fluorescens* SBW25 | viscosin | ViscB mod6 | CAY48788.1 |
|  | Ser | DVWHMSLVDK | *P. fluorescens* SBW25 | viscosin | ViscC mod8 | CAY48789.1 |
|  | Ser | DVWHMSLVDK | *P. fluorescens* Pf-5 | orfamide | OfaB mod6 | AAY91420.2 |
|  | Ser | DVWHMSLVDK | *P. fluorescens* Pf-5 | orfamide | OfaC mod9 | AAY91421.3 |
|  | Ser | DVWHMSLVDK | *Pseudomonas* sp. MIS38 | arthrofactin | ArfB mod6 | BAC67535.1 |
|  | Ser | DVWHMSLVDK | *Pseudomonas sp.* MIS38 | arthrofactin | ArfC mod8 | BAC67536.1 |
|  | Ser | DVWHLSLVDK | *P. putida* | putisolvin | PsoB mod6 | ABW17376.1 |
|  | Ser | DVWHLSLVDK | *P. putida* | putisolvin | PsoB mod9 | ABW17376.1 |
|  | Ser | DVWHLSLVDK | *P. putida* | putisolvin | PsoC mod12 | ABW17377.1 |
|  | Ser | DVWHLSLVDK | *P. entomophila* L48 | entolysin | EtlC M13 | CAK15815.1 |
|  | Ser | DVWHLSLVDK | *P. entomophila* L48 | entolysin | EtlB M10 | CAK15815.1 |
|  | Ser | DVWHLSLIDK | *P. aeruginosa* | pyoverdine | PvdI module 1 &3 | AAX16297.1 |
|  | Ser | DVWHLSLIDK | *P. syringae pv. syringae* | syringopeptin | SypC mod16 | AAO72425.1 |
| **Cyclic** | Pro | DVQYIAHVVK | *P. syringae pv. syringae* | syringopeptin | SypA mod2 | AAF99707.2 |

**Supplementary table 2: Overview of the strains, plasmids and primers used in this study.** Restriction recognition sites are indicated in bold for EcoRI (G^AATTC), PstI (CTGCA^G), HindIII (A^AGCTT) and BamHI (G^GATCC). All primers and chemically-synthesized DNA fragments were obtained from Integrated DNA technologies (Haasrode, Belgium).

|  | **Name** | **Genotype (reference)** |  |
| --- | --- | --- | --- |
| **Strains** | *E. coli* Top10 | F- *mcrA* Δ( *mrr*-*hsd*RMS-*mcr*BC) Φ80*lac*ZΔM15 Δ *lac*X74 *rec*A1 *ara*D139 Δ(*araleu*)7697 *gal*U *gal*K *rps*L (StrR) *end*A1 *nup*G (Invitrogen^TM^) | |
|  | *E. coli* S17-1 | *TpR SmR recA, thi, pro, hsdR-M+RP4: 2-Tc:Mu: Km Tn7 λpir* (Simon *et al.*, 1983) | |
|  | *P. fluorescens* BCCM_ID9359 | / |  |
| **Plasmids** | pUC18 | (Invitrogen^TM^) |  |
|  | pAKE604 | (El Sayed *et al.*, 2003) |  |
| **Primers** | bat2mod1_LIC5’_F | AAA**AAGCTT**GCAGGACAGCGGTGCAC | Amplification of 5' fragment for LIC |
|  | bat2mod1_LIC5’_R | AA**CTGCAG**CCAAGAGCCGGTCATC |  |
|  | bat2synth_F | CCGGCTCTTGGCAGTGAC | Amplification of synthetic DNA fragment |
|  | bat2synth_R | TTGGCCTGCCAATGGAATG |  |
|  | bat2mod1_LIC3’_F | AA**CTGCAG**GCAGGCCAATCGCCAATAC | Amplification of 3' fragment for LIC |
|  | bat2mod1_LIC3’_R | TT**GAATTC**CGTCAGTTGCCGTCGATC |  |
|  | pAKE604_F | GGCTCGTATGTTGTGTGGAATTG | Vector primers for clone analysis |
|  | pAKE604_R | GGGCCTCTTCGCTATTAC |  |
|  | GmR_F | TTT**GGATCC**CCCCTGATTCCCTTTGTC | Amplification of GmR gene |
|  | GmR_R | GTG**GAGCTC**GGCGTTGTGACAATTTAC |  |
|  | bat2mod1_5R | GTG**GGATCC**GAAACTGTAGGTTGTCAC | Reverse primer for 5' fragment amplification for cloning GmR cassette |
|  | bat2mod1_3F | AAA**GAGCTC**CGTCGAAAGAGTCAGTGA | Forward primer for 3' fragment amplification for cloning GmR cassette |
|  | bat2mod1_extF | GTGGAATGCGACAGATG | Amplification of genomic fragment spanning the 5’, synthetic fragment and 3’ |
|  | Bat2mod1_extR | CGACTCGGACGTATCAC |  |

**Supplementary figure 1: Extracted ion chromatograms of culture supernatants from wildtype P. fluorescens BCCM_ID9359 and mutants at expected m/z values.** The m/z value of the WT mass could be detected in the WT supernatant. For the other supernatants, the expected m/z value could not be detected.

**Supplementary figure 2:** **Extracted ion chromatograms at m/z value of WT of culture supernatants from wildtype P. fluorescens BCCM_ID9359 and mutants.** In none of the samples, except for the WT supernatant, the m/z value of 549,3520, corresponding to the WT mass could be detected.
